# Neural Correlates of Growth Mindset: A Scoping Review of Brain-Based Evidence

**DOI:** 10.3390/brainsci15020200

**Published:** 2025-02-14

**Authors:** Hang Zeng

**Affiliations:** Center for Educational Science and Technology, Beijing Normal University, Zhuhai 519087, China; zenghang14@gmail.com

**Keywords:** growth mindset, theory of intelligence, education neuroscience, neuroscience of mindset

## Abstract

Growth mindset, which asserts that intelligence and abilities can be cultivated through effort and learning, has garnered substantial attention in psychological and educational research. While the psychological and behavioral impacts of growth mindset are well-established, the underlying neural mechanisms remain relatively underexplored. Furthermore, there is a lack of comprehensive reviews synthesizing the neural evidence on growth mindset, hindering a fuller understanding of this concept. This scoping review aims to synthesize existing empirical studies on the neural mechanisms of growth mindset, focusing on research objectives, methods, and participant characteristics. A total of 15 studies were reviewed, revealing six primary research objectives: (1) neural mechanisms of error and feedback processing, (2) domain-specific mindsets, (3) neural changes resulting from mindset interventions, (4) mindsets and grit, (5) the neuroanatomy of mindsets, and (6) neural mechanisms of stereotype violation, with error and feedback processing being the most frequently investigated. Ten of the 15 studies employed EEG, while other techniques included structural MRI, task-based fMRI, and resting-state fMRI, with the majority of research focusing on adult populations. Although the existing literature offers valuable insights, further research is needed to explore additional aspects of mindsets, particularly in children, and to refine the methodologies used to investigate the neural mechanisms underlying growth mindset.

## 1. Introduction

The concept of the growth mindset, proposed by Dweck [1,2], has garnered significant attention from researchers, policymakers, and educators. A growth mindset refers to the belief that intelligence and abilities are malleable and can be developed through effort and learning. In contrast, a fixed mindset is characterized by the belief that intelligence is static and cannot be changed. Research on mindsets, or implicit theories of intelligence, has shown that individuals with a growth mindset (incremental theorists) are more likely to embrace challenges [3], persist through setbacks [4], and respond constructively to failure and mistakes [5,6]. These characteristics contribute to higher levels of learning and achievement among incremental theorists [7,8,9]. For instance, Yeager [8] conducted a famous national experiment involving a large, representative sample of 9th graders in the U.S. (N = 12,490), which evaluated the effects of a brief online growth mindset intervention. The intervention significantly improved grades for lower-achieving students and increased the likelihood that students would choose and persist in more challenging math courses compared to the control group.

While the psychological and behavioral effects of growth mindset are well-documented, the neuroscience of growth mindset has recently emerged as an important area of research. Understanding the neural mechanisms that support this psychological construct is essential for a deeper comprehension of how growth mindset influences learning. Early in 2006, researchers began using electroencephalogram (EEG) to investigate how mindsets affect attention to information associated with successful error correction [10]. More recent studies have employed advanced neural imaging techniques, such as resting-state functional magnetic resonance imaging (fMRI) and voxel-based morphometry (VBM), to explore the underlying neural mechanisms of growth mindset [11,12]. However, few comprehensive reviews synthesize the neural evidence on growth mindset, limiting our understanding of this topic. Ng [13] conducted a narrative review focusing on the relationship between growth mindset and intrinsic motivation, examining how neural responses facilitate this relationship. Sarrasin [14] performed a meta-analysis on the effects of growth mindset interventions on motivation, academic achievement, and brain activity, identifying only one study that explored the impact of mindset induction on brain activity [15]. The authors nevertheless included studies on the neural mechanisms associated with growth mindset as a trait, not an intervention [5,10,12,16]. However, no updated review has synthesized the recent findings on the neural mechanisms of growth mindset, leaving gaps in the current literature.

Understanding the neural mechanisms underlying growth mindset is essential for explaining how this mindset influences learning outcomes and how individuals with different mindsets engage with complex tasks. The aim of this paper is to provide a scoping review of existing empirical studies on the neural mechanisms related to growth mindset. Scoping reviews are particularly valuable for mapping emerging evidence and identifying research gaps [17]. This approach can inform future research agendas and guide the development of interventions aimed at enhancing growth mindset. Specifically, this scoping review seeks to address the following five research questions:

RQ1: What objectives and outcomes are reported in the included studies?

While the overarching theme of the selected studies is neural mechanisms related to growth mindsets, each study focuses on specific aspects. This question aims to provide a comprehensive view of the specific research objectives and evidence found in these studies.

RQ2: What techniques and neural measurements are used in the included studies?

This question seeks to identify the types of techniques (e.g., EEG, fMRI) and related neural measurements (e.g., Pe, ERN, gray matter volume, BOLD signal) employed by researchers to investigate the neural mechanisms related to growth mindset.

RQ3: What are the sample sizes, regions, and age demographics of the populations in the included studies?

This question aims to identify the most frequently investigated populations, their age groups, and the sample sizes in these studies.

## 2. Methods

This scoping review has been registered with the Open Science Framework (OSF). The protocol for this review was developed according to the Preferred Reporting Items for Systematic Review and Meta-Analysis (PRISMA) extension for scoping review [18] and can be accessed at https://doi.org/10.17605/OSF.IO/EQUJ8 (accessed on 11 February 2025). Below, I report the inclusion and exclusion criteria, the literature search, the literature selection, and the data coding and extraction.

### 2.1. Inclusion and Exclusion Criteria

I established specific inclusion and exclusion criteria to guide our selection of relevant literature, as detailed in Table 1. I considered original research articles, including peer-reviewed journal articles and conference papers. Articles that were not original research, including literature reviews, meta-analyses, editorials, book reviews, reports, and preprints that had not undergone peer review, were excluded. The focus of the literature had to be on investigating neural activity, neural mechanisms, or brain signals of growth/fixed mindset or implicit theory of intelligence. Studies that focused solely on behavioral aspects without a neural component, as well as those that examined mindsets unrelated to attitudes toward abilities and intelligence, were excluded. Studies could have been conducted in any country, but only English-language articles were included. I did not impose any time range limits on publication dates and included studies with participants from all age groups.

### 2.2. Literature Search

First, I performed an electronic search in the following core databases for neuroscience, psychology, and education—Web of Science, EBSCOhost (including Academic Search Ultimate, APA PsycTests, Psychology and Behavioral Sciences Collection, ERIC, MEDLINE, and OpenDissertations), ProQuest (including APA PsycInfo, ProQuest Dissertations and Theses Global, Psychology Database, Education Database, and APA PsycArticles^®^). The search was conducted in January of 2025. The search string in Web of Science was (TS = (mindset OR implicit theory of intelligence)) AND TS = (neural mechanism OR neural activity OR neural correlate OR brain signal OR brain activity OR neuroscience OR neuroanatomy OR cortex). Language and article types were further added as search criteria. I fine-tuned our search string through several trial searches to make sure the search string was neither so specific that some relevant articles were missed nor so broad that the number of hits was manageable in later steps. For example, “mindset” instead of “growth mindset OR fixed mindset” was used to include studies that investigate subcategories of mindsets, such as mathematical mindsets. The search strings for the other databases were the same, but the formats were adapted to each database.

### 2.3. Literature Selection

**Figure 1:**
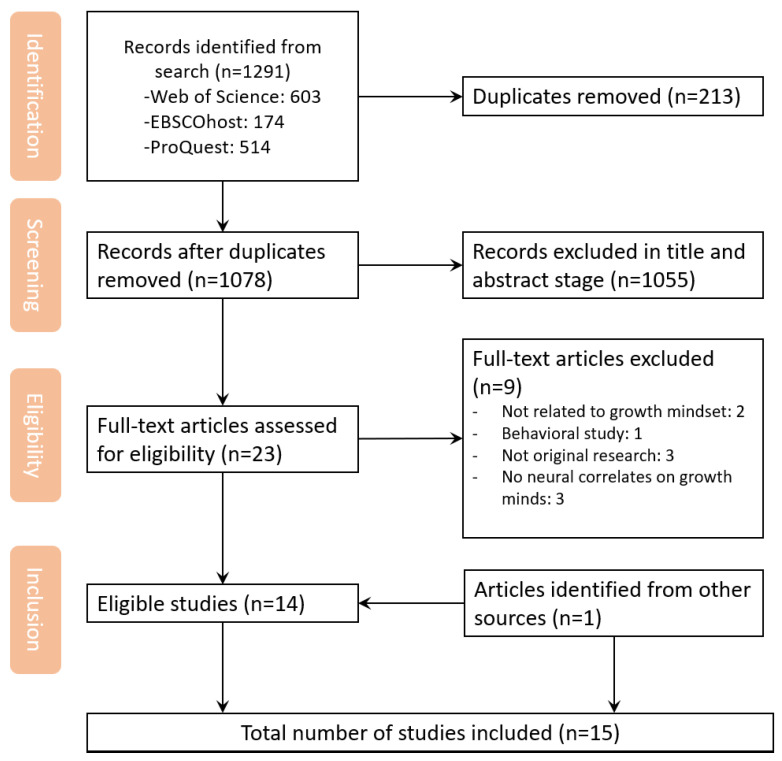
Study selection flow diagram.

Two reviewers independently screened the studies for eligibility in a hierarchical approach. The identified articles were managed in Zotero. First, the reviewers screened the titles and abstracts identified in the databases to identify potential articles. Second, the reviewers screened the full-text articles. Studies that did not meet the eligibility criteria were removed. Third, I performed the backward and forward search process. Additionally, I checked the reference list of each included article to find other articles that were not included in the database search but could potentially be eligible for inclusion in the review. Lastly, I reviewed the reference lists of previous relevant reviews. Throughout this process, any discrepancies were resolved in a discussion until a consensus was achieved. The selection process is displayed in a PRISMA flowchart (see Figure 1).

### 2.4. Data Coding and Extraction

Two raters coded the selected studies independently using a systematic coding form (see Table 2). Specifically, publication characteristics and information regarding each research question were extracted from the selected papers. Again, potential discrepancies were resolved in a discussion until a consensus was reached.

## 3. Results

### 3.1. Search Results

The search initially identified 1291 articles, which were reduced to 1078 after removing duplicates. We further excluded 1055 articles after reviewing the titles and abstracts for eligibility, resulting in 23 articles. Fourteen articles were eligible for inclusion after full-text assessment. One additional eligible article was identified from other resources. In summary, a total of 15 articles were included in this scoping review (see Figure 1).

### 3.2. Description of Included Studies

The included studies were published between 2006 and 2023 (see Table 3), with 9 out of the 15 papers published since 2018. The total sample size was 1374 participants, considering only those whose neural activities were recorded. All studies were published in peer-reviewed journals.

### 3.3. What Objectives and Outcomes Are Reported in the Included Studies?

**Figure 2:**
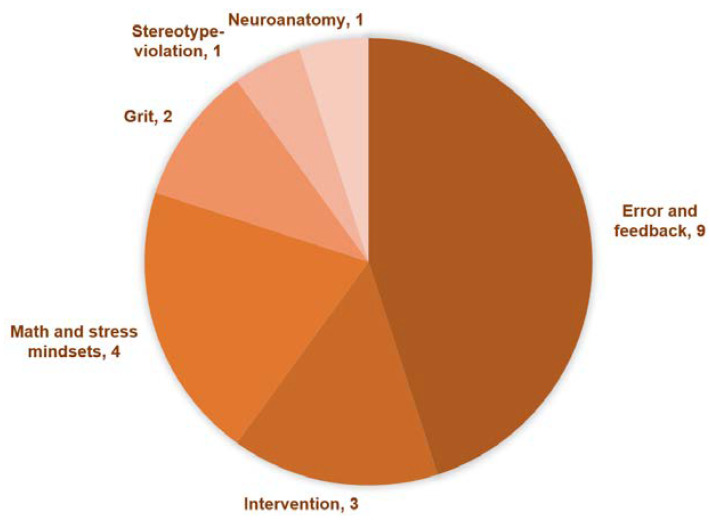
Research objectives of selected papers.

After analyzing the 15 papers, 6 types of objectives were classified, and some papers covered more than one objective (see Figure 2).

#### 3.3.1. Neural Mechanisms Underlying Error and Feedback Processing of Mindsets

Nine studies investigated the neural mechanisms underlying error and feedback processing of mindsets, making it the most frequently explored topic [5,10,12,15,16,19,20,21,22]. Error and response feedback processing can be seen as two variants of feedback: one concerned with the processing of internal feedback, and the other with external feedback [23].

Mangels [10] was the first to investigate the neural mechanisms of the growth mindset, using EEG to study the brain activity of 47 undergraduate students as they answered general knowledge questions across various academic disciplines. Based on Dweck’s Theory of Intelligence scale, participants were categorized as having either a growth (incremental theorists) or fixed mindset (entity theorists). During the test, participants received positive or negative feedback on their answers, followed by a surprise retest on initially incorrect responses. Entity theorists showed enhanced anterior frontal P3 responses to negative feedback, indicating heightened cognitive engagement and a focus on proving their abilities. However, after receiving correct answers, they exhibited reduced sustained memory-related activity (left temporal negativity), suggesting less effortful conceptual encoding. This processing bias likely contributed to their poorer error correction on the retest. These findings highlight how beliefs about intelligence can shape attention and learning by top-down biasing toward goal-congruent information.

Moser [5] later supported these findings, examining the neural mechanisms by which growth and fixed mindsets influence error monitoring and behavioral adjustments following mistakes. The authors aimed to extend the findings of Mangels [10] by examining response-locked ERPs that tap into internal performance-monitoring processes elicited by response execution in a speeded reaction time (RT) task. Using a sample of 25 undergraduate students, researchers assessed mindsets through the Theory of Intelligence scale and measured neural activity using event-related potentials (ERPs) during a letter version of the Eriksen Flanker task. The error-related negativity (ERN) and error positivity (Pe) components of the ERPs were analyzed, as they reflect cognitive control and attention allocation to errors, respectively. Results revealed that participants with a growth mindset exhibited enhanced Pe amplitudes, suggesting heightened error awareness and attentional focus on mistakes, which mediated improved post-error accuracy. In contrast, fixed-mindset individuals showed weaker Pe responses and less adaptive behavioral adjustments. The findings demonstrate that growth mindsets promote more effective error processing by enhancing neural attention to mistakes, which supports better learning and task performance.

Schroder [16] replicated the adult work of Moser [5] with 123 6-to-8-year-old children. They investigated the relationship between growth mindset, neural error monitoring, and post-error performance, using a go/no-go task. Results revealed that children with a growth mindset demonstrated greater Pe amplitudes, indicating enhanced attention allocation to mistakes. These findings extend prior adult research, indicating that even young children with a growth mindset show improved resilience to errors via enhanced neural processing and behavioral adjustments. The study emphasizes the potential of fostering growth mindsets early in development to support adaptive responses to mistakes in educational contexts.

The same research group also examined how experimentally induced growth or fixed mindsets influence cognitive control/and associated neural processes [15]. Forty-four undergraduate participants were randomly assigned to read scientific articles presenting intelligence as either malleable (growth mindset) or fixed (fixed mindset) before completing the same Eriksen Flanker task as in Moser [5], a reaction-time task designed to elicit errors and cognitive control processes. EEG was used to measure brain activity, focusing on ERPs such as the N2, P3, ERN, and Pe. Results showed that participants in the growth mindset condition exhibited enhanced P3 amplitudes, reflecting greater attentional allocation to stimuli and adaptive behavioral adjustments after errors, as indicated by reduced post-error slowing (PES) and improved post-error accuracy (PEA). In contrast, participants in the fixed mindset condition demonstrated enhanced late Pe amplitudes, reflecting greater attention to responses, but these neural responses were not linked to behavioral improvements. These findings suggest that growth mindsets promote more adaptive cognitive control strategies, emphasizing the importance of mindset inductions in fostering efficient error monitoring and recovery. Importantly, in contrast to Moser [5] and Schroder [16], they observed an enhanced Pe to the response for the fixed mindset group. They argued that the enhanced attention to responses in the fixed condition did not confer a behavioral advantage on subsequent trials. However, early Pe difference amplitudes in the growth condition were associated with faster and more accurate responses after errors (reduced PES and enhanced PEA). Thus, the brain–behavior relationship patterns suggest that early attention allocation to errors was directly associated with efficient post-error behavior in the growth condition only, which converged with their study of trait mindsets [5,16].

Using resting-state fMRI, Myers [12] further investigated the cerebral mechanisms associated with different mindsets. The study involved twenty 11-year-old children, whose mindsets were assessed using a questionnaire before conducting resting-state fMRI connectivity analysis. The results revealed that students with a growth mindset exhibited higher connectivity in cortico-striatal pathways, specifically between (1) the dorsal striatum and (2) the dorsal anterior cingulate cortex and dorsolateral prefrontal cortex. According to the authors, this suggests that the neural networks associated with a growth mindset include regions involved in regulation strategies and error-monitoring. These findings align with prior EEG research, highlighting that individuals with a growth mindset demonstrate enhanced connectivity in brain regions related to attentional resources and the use of positive strategies.

Pursuing the same question, Janssen [20] aimed to investigate the relationship between intelligence mindset (growth vs. fixed) and error-monitoring mechanisms using EEG, with a specific focus on addressing a critical methodological concern: stimulus-response overlap. The researchers emphasized the need to control for this overlap because it could confound neural measures. For example, Pe may even be a delayed P3 related to stimulus processing rather than error-monitoring. They tested 89 participants with a Stop-Signal Task (SST) and targeted four ERPs: stop-stimulus-locked P3 component, response-locked Ne and Pe components, and stimulus-locked N2 component. Importantly, they corrected for stimulus-response overlap using Adjacent Response Convolution Analysis (ADJAR). Results showed that behavioral measures such as post-error slowing and accuracy were unrelated to mindset. Importantly, while initial results showed larger Pe amplitudes in fixed-mindset individuals, this effect disappeared after correcting for stimulus-response overlap, indicating that these differences were likely driven by delayed stimulus-locked P3 rather than genuine error evaluation. The findings challenge previous assumptions about the neural correlates of mindset, highlighting the complexity of error-monitoring mechanisms. Authors called for future studies to control for stimulus-response overlap and examine broader contextual and individual factors influencing error monitoring and mindset.

Bejjani [19] investigated how intelligence mindset (growth vs. fixed) influences behavioral and neural responses to feedback during learning, particularly under a competence threat. Forty participants completed a fake intelligence test and were assigned to either a competence-threat condition (low IQ score) or a non-threat condition (no score). They then performed a paired-associate word-learning task with two feedback conditions: blocked feedback (predictable, emphasizing evaluative weight) and mixed feedback (intermittent, emphasizing informational weight). The design was intended to assess how mindset affects the perception and utilization of feedback in contexts where it is more evaluative or more informative. Behavioral and neural responses were assessed using test accuracy and fMRI data focused on the caudate nucleus. Results showed that competence threats impaired learning for fixed-mindset individuals (entity theorists), who interpreted negative feedback as punitive and exhibited inflexible striatal responses. Specifically, fixed-mindset individuals exhibited stronger “punishment” responses to negative feedback in the caudate nucleus, particularly after a competence threat, and failed to benefit from feedback in the evaluative (blocked) context. Growth-mindset individuals showed more flexible striatal responses, with greater activation to mixed feedback, reflecting their ability to interpret negative feedback as informative. These findings highlight the impact of mindset on feedback processing, suggesting that fixed mindsets amplify the negative effects of competence threats on learning and memory, while growth mindsets support more adaptive learning strategies.

Focusing on the math mindset, Puusepp [21] examined general intelligence and math ability mindsets and their relations to automatic reactions to negative feedback in mathematics in elementary students. Neural activities of 97 third graders were measured during an age-appropriate math task that involved solving equations and receiving performance feedback. Feedback was presented visually for correct responses, while incorrect responses included both a feedback tone and corrective information to ensure clarity. They found that a higher growth mindset was marginally associated with a larger P300 response to negative feedback and significantly linked to a smaller, later-peaking negative-going waveform. This finding is in contrast to Mangels [10], who identified a greater frontal P300 associated with a fixed mindset and performance goal endorsement, indicating the salience of negative performance feedback for fixed-minded individuals. Authors argued that frontally maximal P300 in Mangels [10] was elicited by performance-relevant feedback stimulus, but in their study, performance-relevant feedback was presented simultaneously with corrective feedback. Thus, in this case, a larger P300 could indicate more attentional resources engaged in the processing of the corrective feedback stimulus for the growth mindset. Furthermore, using a domain-specific experimental design, the study found that a higher growth mindset about math ability—but not general intelligence—was associated with these brain responses to negative feedback related to math errors. These results suggest that mindsets regarding specific domains possibly play a bigger role in eliciting automatic reactions to feedback in the corresponding domains when compared to more general mindsets.

Puusepp [22] conducted a follow-up study a year later. They examined the development of the associations between elementary school students’ mindsets and the attentional neural processing of positive and negative feedback in math. Participants, 100 students from grades 3 and 4, completed an age-appropriate arithmetic task similar to their previous study. Neural responses were recorded via EEG, focusing on the P300 component, which reflects attentional resource allocation to feedback. Results showed that a more fixed general intelligence mindset in 4th graders was marginally associated with greater attention to negative feedback. Additionally, students with fixed mindsets about general intelligence and math ability showed greater attention to positive feedback, reflected in larger P300 amplitudes, particularly in grade 4. The latter finding diverges from Mangels’ results [10], which found no such association. One potential explanation for this difference is the task relevance to participants’ daily lives. While Mangels [10] used general knowledge questions with undergraduates, their study focused on math, a subject highly relevant to elementary school students. Self-relevant stimuli tend to capture more attention, as shown by larger P300 responses, which may explain why students with a fixed mindset allocated more attention to feedback. Another possibility is that feedback may be perceived as more threatening for fixed-minded individuals, leading to a larger P300 response due to its higher perceived magnitude. Developmental trends suggest that the influence of mindset on attentional feedback processing strengthens with age, likely due to increasing self-relevance of feedback. These findings underscore the importance of fostering growth mindsets early to support attentional processing and enhance academic outcomes.

In summary, these studies highlight the relationship between mindsets, learning goals, attention to errors, and post-error behavior. Individuals with a fixed mindset, focused on performance goals, tend to pay more attention to external feedback, but this does not improve post-error strategies or accuracy. In contrast, those with a growth mindset, driven by learning goals, allocate more attention to the stimulus or errors (internal feedback, instead of negative external feedback), leading to better memory encoding, improved error correction, and higher achievement through positive strategies. However, mixed findings and potential limitations related to stimulus-response overlay warrant further investigation to provide a more comprehensive understanding.

#### 3.3.2. Neural Mechanisms of Specific Domain of Mindsets (Math and Stress)

Four studies focused on specific domains of mindsets, including math mindset [21,22,24] and stress mindset [25]. Two of the math studies, which also cover the topic of feedback, have been introduced above.

**Table 3:** Information on the included papers.

No	Authors	Year	Publication Type	Region	Sample Size	Participant Age	Experimental Techniques
1	Bejjani et al. [19]	2019	Journal Paper	USA	40	23.48 ± 6.06	Task-based fMRI
2	Chen et al. [26]	2022	Journal Paper	USA	79	8.20 ± 0.65	Task-based fMRI
3	Daly et al. [24]	2019	Journal Paper	UK	16	20 ± 0.74	EEG
4	Janssen et al. [20]	2021	Journal Paper	Netherlands	89	20.9 ± 1.9	EEG
5	Jia et al. [11]	2023	Journal Paper	China	389	19.39 ± 1.17	Structural MRI/VBM
6	Mangel et al. [10]	2006	Journal Paper	USA	47	Entity group: 21.0 ± 0.64, Incremental group: 21.6 ± 0.56	EEG
7	Moser et al. [5]	2011	Journal Paper	USA	25	20.25	EEG
8	Myers et al. [12]	2016	Journal Paper	USA	20	11.2	Resting-state fMRI
9	Park et al. [25]	2019	Journal Paper	South Korea	30	Mainly from 18 to 24	EEG
10	Puusepp et al. [22]	2023	Journal Paper	Finland	100	8.94 ± 0.43	EEG
11	Puusepp et al. [21].	2021	Journal Paper	Finland	97	8.94 ± 0.43	EEG
12	Schroder et al. [15]	2014	Journal Paper	USA	44	19.70 ± 1.46	EEG
13	Schroder et al. [16]	2017	Journal Paper	USA	123	6.01 ± 8.26 years	EEG
14	Wang et al. [27]	2018	Journal Paper	China	231	18.48 ± 0.54	Structural MRI/VBM
15	Xu et al. [28]	2015	Journal Paper	Canada	44	18.21 ± 2.61	EEG

Daly [24] investigated whether mathematical problems formulated according to mathematical mindset (MM) theory can increase learner motivation and explore the neural correlates of this effect. Sixteen participants engaged with both standard and MM-formulated problems while their motivation was measured through self-reports and prefrontal EEG asymmetry, a neural marker of motivation. The MM-formulated problems were structured in ways that encouraged learners to engage with the problems, not only to find the correct answers but also to approach the problem-solving process as an opportunity for growth and learning. Results showed that learners found MM problems significantly more motivating than standard problems, with corresponding differences in prefrontal EEG asymmetry, indicating distinct brain activity related to motivation. This study provides early evidence that MM-based problems can enhance short-term motivation in learners, even when they are not explicitly told about the mindset theory, and highlights the neural mechanisms underlying this motivational shift.

One study investigated the stress mindset [25]. They evaluated the effectiveness of an online educational intervention in shifting individuals’ stress mindsets from “stress-is-debilitating” (SDM) to “stress-is-enhancing” (SEM) and examined the associated neural and behavioral changes. The study involved 479 college students who participated in a series of three 15-min online sessions over 16 days. These sessions included video-based education about the concept of stress mindsets, the relationship between stress responses and hormones, and emotional storytelling featuring examples of successful individuals who benefited from an SEM. Stress mindset was measured using a validated 10-item questionnaire, and EEG data were collected from a subgroup of 30 students before and after the intervention, focusing on brain activity during stress-inducing interviews. Results showed a significant increase in mindset scores, with the average shifting from 3.38 to 4.15 on a 7-point scale (*p* < 0.001). EEG findings revealed increased alpha wave activity and reduced high-beta wave activity post-intervention, indicating a healthier and more adaptive neural response to stress. Additionally, participants reported improved attitudes and coping strategies in stressful situations. The study highlights the potential of scalable e-healthcare programs to promote positive stress mindsets and improve stress resilience, with implications for application in schools, workplaces, and healthcare settings.

#### 3.3.3. Neural Changes of Mindset Intervention

Three studies examined the neural changes associated with mindset interventions. Schroder [15] induced a growth mindset by having participants read an article suggesting that intelligence can be developed through effort, while a fixed mindset was induced by an article claiming intelligence is largely determined by genetics and cannot be changed. Details of this study are provided in Section 3.3.1. Park and Hahm [25] implemented a stress mindset intervention, consisting of three 15-min online video sessions over 16 days, which focused on educating participants about stress mindsets. Further details of this study are provided in Section 3.3.2.

Chen [26] examined the impact of a four-week tutoring-based cognitive training program on growth mindset and its neural mechanisms in children aged 7–10. The training program was a four-week, one-on-one tutoring-based program, focused on fundamental numerical concepts (e.g., counting, comparing, ordering). It emphasized mastery-oriented learning with positive feedback. The training group exhibited significant gains in growth mindset compared to the control group, with the most substantial improvements seen in children with lower initial growth mindset. fMRI analysis revealed that growth mindset improvements were associated with increased activation and functional connectivity in the dorsal anterior cingulate cortex, striatum, and hippocampus, highlighting the role of cortico-striatal circuits in growth mindset malleability. These findings suggest that cognitive training can enhance both academic skills and growth mindset through neural plasticity, offering valuable insights for interventions targeting children with learning difficulties.

#### 3.3.4. Mindsets and Grit

Given the close relationship between mindset and grit, two studies investigated the neural mechanism of mindset and its relation to grit.

Myers [12] investigated the similarities and differences of neural mechanisms underlying grit and growth mindset using resting-state fMRI in twenty children. They found that grit, the long-term perseverance towards a goal or set of goals, was associated with ventral striatal networks, including connectivity to regions such as the medial prefrontal and rostral anterior cingulate cortices implicated in perseverance, delay, and receipt of reward. Growth mindset, the belief that effort can improve talents, notably intelligence, was associated with both ventral and dorsal striatal connectivity with regions thought to be important for error-monitoring, such as dorsal anterior cingulate cortex.

Wang [27] investigated the relationship between grit and brain structure, and the mediating role of growth mindset in this association. Using VBM, they analyzed the neuroanatomical correlates of grit in 231 healthy adolescent students through structural MRI. Whole-brain regression analyses revealed that regional gray matter volume (rGMV) in the left dorsolateral prefrontal cortex (DLPFC) was negatively associated with grit, while rGMV in the right putamen showed a positive association with grit. Moreover, mediational analyses indicated that growth mindset mediated the relationship between left DLPFC volume and grit. These findings remained robust even after accounting for the influences of self-control and delayed gratification. In conclusion, the study offers novel evidence for the neuroanatomical basis of grit and underscores the potential role of a growth mindset in fostering a student’s level of grit.

#### 3.3.5. Neuroanatomy of Mindsets

One study directly investigated the neuroanatomy of mindsets. Jia [11] used VBM to examine the relationship between growth mindset and gray matter volume in a sample of 389 Chinese adults (114 male and 275 female), all of whom completed the Growth Mindset Inventory and an intelligence test and underwent structural MRI scans. Whole-brain correlation analyses revealed a positive association between growth mindset scores and regional GMV in the medial orbitofrontal cortex (mOFC), even after controlling for age, sex, and total intracranial volume. This result remained robust after adjusting for intelligence quotient. Given that the mOFC is primarily involved in reward processing, authors suggest that findings support the social-cognitive theory of motivation in the context of growth mindset.

#### 3.3.6. Neural Mechanism of Stereotype-Violation

One study investigated whether different people with a fixed or growth mindset would display systematically different neurophysiological responses to stereotype violating versus stereotype-confirming information. Xu and Plaks [28] used EEG to record brain activity while participants read a series of stereotype-confirming or stereotype-violating behaviors performed by a target character. Specifically, participants read a booklet with a vignette about “Brad”, a first-year undergraduate who was portrayed as strong in math and sciences but weak in arts and humanities. Brad agreed to take an intensive remedial course to improve his writing and literature skills, indicating both the opportunity and motivation for self-improvement. Participants were then presented with 41 stereotype-consistent behaviors and 42 stereotype-inconsistent behaviors. For example, stereotype-inconsistent information involved Brad disliking math or excelling in humanities, while stereotype-consistent information showed his preference for math and sciences. Brain activity was recorded as the sentences were presented. The results indicated that entity theorists exhibited a marginally stronger N400 response (an ERP component associated with social expectancy violations) to stereotype-violating information, whereas incremental theorists did not. This suggests that entity theorists, with their more rigid views on trait fixedness, perceived stereotype-violating behavior as especially incongruent. In contrast, and as predicted, incremental theorists displayed more pronounced N400 responses to stereotype-confirming information compared to entity theorists. This suggests that for incremental theorists, behavior that is “too consistent” is perceived as particularly incongruous.

### 3.4. What Techniques and Neural Measurements Are Used in the Included Studies?

**Figure 3:**
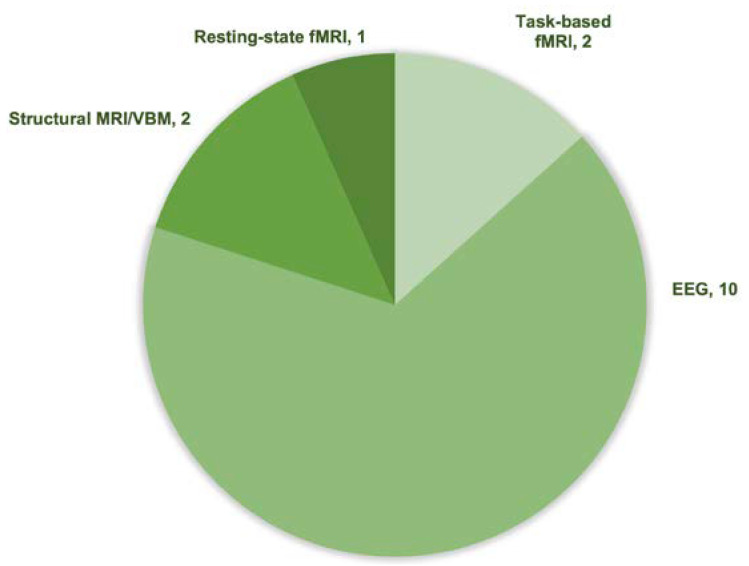
Information on techniques used in the included studies.

Out of the 15 included studies, 10 employed EEG recordings to measure neural activity (see Table 3 and Figure 3), with 8 using ERP, making it the most common technique. Five studies used MRI to assess neural activity. Specifically, two studies utilized structural MRI and VBM to measure gray matter volume associated with different mindsets [11,27]. One study used resting-state fMRI to investigate striatal resting-state connectivity and its correlation with mindset [12], while two task-based fMRI studies examined BOLD signal changes in response to feedback: one in the caudate under competence threat [19] and another in regions linked to cognitive control, motivation, and memory in response to growth mindset [26]. Detailed neural measurements and key findings are summarized in Table 4 and Table 5. Below, I focus on neural measurements related to feedback and error processing across different mindsets.

Specifically, seven studies investigated the topic of feedback or error processing, and the neural measurements they investigated included N2, P300 (P3), ERN, Pe, FRN, and Left Temporal Negativity. The cognitive meanings of these ERPs are summarized in Table 4. Studies showed that mindsets had no effect on N2 amplitudes [15,20], ERN amplitudes [5,15,16,20], and FRN [21], suggesting that mindsets are not related to the initial detection of the outcome valence itself and cognitive control in trials following errors or conflict-related processes. Mangel [10] demonstrated that entity theorists showed reduced left temporal negativity after receiving corrective feedback, indicating less effortful encoding of corrective information.

Studies investigating P300 in error and feedback processing showed that participants with fixed mindsets showed greater attention to negative feedback, as indicated by larger P300 amplitudes in adults [10] and in 4th graders [22], but Puusepp and colleagues [21] found that a higher growth mindset was marginally associated with a larger P300 response to negative feedback in 3rd graders. Schroder [15] used a letter version of the Eriksen Flanker task without providing feedback and found that inducing a growth mindset resulted in enhanced attention to task-relevant stimuli (indicated by enhanced P3) than the fixed mindset.

Lastly, studies that investigated Pe components demonstrated mixed patterns. Both Moser [5] and Schroder [16] found that a growth mindset was related to greater attention to mistakes, as indicated by larger Pe amplitude, but Moser [5] investigated adults and found that Pe mediated the link between growth mindset and post-error accuracy, though not in the children studied by Schroder [16]. Authors suggest that more attention allocation to errors (Pe) may be a necessary mechanism linking the growth mindset belief with the tendency to bounce back from mistakes. In contrast, attention allocation is not necessary for growth-minded children to bounce back. However, when mindsets were induced through brief manipulation in adults, Schroder [15] found that fixed-mindset individuals showed a larger Pe to mistakes, in contrast to Moser [5] and Schroder [16]. Lastly, Janssen [20] addressed the limitation of potential overlap of stimulus- and response-related processing, and found that larger Pe amplitudes were initially observed in fixed-mindset individuals but disappeared after correcting for stimulus-response overlap.

### 3.5. What Are the Sample Sizes, Regions, and Age Demographics of the Populations in the Included Studies?

Sample sizes with neural data collected were analyzed. Four studies recruited 30 participants or fewer, with the smallest sample size being 16 students. Eight studies had sample sizes ranging from 40 to 100. Only three studies recruited more than 100 participants, with the largest sample size being 389 (see Table 3).

In terms of participant demographics, the majority of studies focused on adults, with 10 studies involving primarily university students with an average age of 18 to 20. Five studies targeted elementary school students with an average age of 6 to 11 (see Table 3). Geographically, seven studies were conducted in the USA, two in China, and two in Finland, with the remainder taking place in Canada, the Netherlands, South Korea, and the UK (see Table 3).

## 4. Discussion

This study examines the neural evidence of growth mindset through a scoping review of 15 studies. Notably, nine of the 15 studies were published since 2018, highlighting the recent surge of interest in this area. Various neural recording and neuroimaging techniques (e.g., EEG, functional and structural MRI) have been used to investigate the neural basis of the Growth Mindset. EEG studies have focused on ERN, Pe, and P300 components, linking them to adaptive responses to error processing and learning. MRI studies highlight the role of the prefrontal cortex (e.g., dorsolateral prefrontal cortex, anterior cingulate cortex) and reward-related regions, suggesting that Growth Mindset is associated with neural processes related to cognitive control, motivation, and adaptive learning. Importantly, our review highlights that the neural correlates of Growth Mindset, such as ERN or activity in the dorsolateral prefrontal cortex, are not static markers of a fixed trait but rather reflect transient cognitive states that fluctuate depending on task demands, learning experiences, and environmental cues. While these studies have advanced our understanding of the neural mechanisms underlying growth mindset, several key concerns in the field remain to be addressed.

### 4.1. Limited Topics Investigated

The most extensively studied topic is error and feedback processing in relation to mindset. However, other important aspects of mindsets, such as challenge-seeking behaviors, attitudes towards effort, and persistence through setbacks, remain underexplored. Additionally, Puusepp [21] found that a growth mindset specifically about math ability—not general intelligence—was linked to distinct brain responses to negative feedback related to math errors. This suggests that mindsets regarding specific domains may have a more significant impact on automatic feedback reactions within those domains compared to more general mindsets. Previous studies have shown that one may hold growth beliefs in one domain and fixed beliefs in a different domain [29,30]. Future studies should examine whether the findings of Puusepp [21] can be replicated across other discipline-specific mindsets, which could inform the development of more effective, domain-specific mindset interventions. Another limitation is the insufficient consideration of individuals’ psychological status when investigating neural correlates of Growth Mindset. Evidence suggests that anxiety and panic can significantly modulate P300 amplitudes [31]. These psychological factors can modulate attentional responses to external stimuli, potentially confounding the interpretation of Growth Mindset-related neural activity. Future research should incorporate assessments of psychological status to provide a more nuanced understanding of Growth Mindset’s neural correlates.

### 4.2. Mixed Findings

Mixed findings have emerged in studies investigating the same research question. For example, both Moser [5] and Schroder [16] found that a growth mindset was associated with greater attention to mistakes, as indicated by larger Pe amplitudes. However, when mindset was manipulated briefly in adults, Schroder [15] found that the fixed mindset group demonstrated a larger Pe response to mistakes. Could this be attributed to differences between trait and state mindsets? While one might expect that trait and state mindsets induced by brief interventions would produce similar effects, this discrepancy suggests the need for further investigation. Future studies should directly compare the neural mechanisms underlying trait and state mindsets to provide a more comprehensive understanding.

Additionally, while Mangel [10] found that participants with fixed mindsets showed greater attention to negative feedback, indicated by larger P300 amplitudes in adults, Puusepp [21] found that a higher growth mindset was marginally associated with a larger P300 response to negative feedback in 3rd graders. The authors argued that, in their study, performance-relevant feedback was presented alongside corrective feedback, and that a larger P300 response could reflect greater attentional resources dedicated to processing the corrective feedback stimulus for the growth mindset group. However, in a similar experiment, Puusepp [22] found that a larger P300 response was associated with negative feedback in the fixed mindset group in 4th graders. Specifically, in grade 4, greater P300 amplitudes were elicited by negative feedback compared to positive feedback—an effect not observed in 3rd graders. The authors suggested that this reflects the developmental increase in the salience of negative performance feedback in math relative to positive feedback. More studies focusing on the developmental processes of mindsets are needed to clarify this issue.

Importantly, Janssen [20] addressed a critical methodological concern regarding stimulus-response overlap. They found that while initial results indicated larger Pe amplitudes in fixed-mindset individuals, this effect disappeared after correcting for stimulus-response overlap. These findings challenge previous assumptions about the neural correlates of mindset, particularly the belief that individuals with a fixed mindset allocate more attention to negative feedback. Future studies should take stimulus processing into account and, when necessary, correct for stimulus-response overlap to ensure more accurate conclusions.

### 4.3. Lifespan Perspectives on Growth Mindset

Two-thirds of the included studies investigated adults, leaving only one-third focused on school-aged children. While research on adults provides valuable insights, children’s brains are still developing, which likely leads to different neural responses to mindset-related interventions compared to adults. Understanding the neural processes of growth mindset in children could offer important insights into how mindsets can be cultivated during critical stages of cognitive and emotional development [32]. Children are more malleable in terms of their beliefs and brain structures, making them an ideal population for studying the impact of mindset on neural plasticity. By exploring how a growth mindset affects brain areas related to learning, motivation, and resilience, researchers could identify ways to tailor interventions that foster these qualities at earlier stages of development [33]. Furthermore, studying the neural mechanisms in children could provide a better understanding of the long-term effects of growth mindset interventions, which may enhance educational strategies and support more effective interventions in schools. More research is needed to explore the neural mechanisms of growth mindset in children. Lastly, understanding Growth Mindset in older adults is equally important, particularly for identifying neural correlates that may contribute to cognitive resilience and healthy aging. Future research should explore how cultivating a Growth Mindset might influence mental and cognitive health across the lifespan, potentially offering pathways to support healthy longevity.

## 5. Conclusions

I conducted a scoping review of the neural mechanisms underlying growth mindset, synthesizing findings from 15 studies that addressed our research questions. I identified the primary objectives and outcomes of these studies, the techniques and neural measurements employed, and summarized the sample sizes, participant demographics including regions and age ranges.

Regarding the main objectives, six objectives were identified, including (i) neural mechanisms underlying error and feedback processing of mindsets; (ii) neural mechanisms of specific domain of mindsets (math and stress); (iii) neural changes of mindset intervention; (iv) mindsets and grit; (v) neuroanatomy of mindsets; and (vi) neural mechanism of stereotype-violation. Regarding research techniques, ten studies utilized EEG recordings to measure neural activity, with ERPs related to error and feedback processing being the most commonly used neural measurement. As for the participant population, the majority of studies focused on adults, with sample sizes typically ranging from 40 to 100 participants.

Overall, while the existing body of research offers valuable insights, there is a need for more studies exploring additional important aspects of mindsets, particularly those focusing on children, to further our understanding of the neural mechanisms associated with growth mindset.

## Figures and Tables

**Table 1:** Criteria for inclusion and exclusion in the scoping review.

Criterion	Inclusion	Exclusion
Article type	Original research, including peer-reviewed journal articles and conference papers	Articles that were not original research (e.g., literature reviews, meta-analyses, editorials, book reviews, or reports) or preprint papers
Literature focus	Investigate the neural activity/neural mechanism/brain signal of growth/fixed mindset or implicit theory of intelligence	Behavioral studies, other types of mindsets that were not related to growth mindset
Language	English	Non-English
Publication date	Until 2025	-
Age group	All age groups	-

**Table 2:** Data coding and extraction form.

Characteristics	Information	Relevant RQ
Author	Name of the first author	Publication characteristics
Publication year	The time when the article was published	Publication characteristics
Publication type	Peer-reviewed journal article, or conference paper	Publication characteristics
Objectives	Purpose of the selected study	RQ1
Outcomes	Main neural and behavioral findings	RQ1
Experimental techniques	EEG, MRI, or other techniques	RQ2
Neural measurements	Pe, ERN, gray matter volume, or other measurements	RQ2
Age	Age of participants	RQ3
Region	Region of participants recruited from	RQ3
Sample size	Sample sizes of neural data collected	RQ3

**Table 4:** Information on neural measurements and related findings of included EEG studies.

Measures	Cognitive Meaning	Main Neural Findings Regarding Mindsets
N2	- Cognitive control and response inhibition (e.g., go/no-go tasks). - Conflict detection and resolution. Time: 200–350 ms post-stimulus.	- Mindset had no effect on N2 amplitudes [15,20]
P300 (P3)	- Attention allocation to task-relevant or unexpected stimuli. - Reflects cognitive processing of important stimuli. Time: Around 300 ms after stimulus presentation.	- Entity theorists responded to negative feedback with heightened anterior frontal P3 activity, reflecting concerns about proving ability [10]
		- Inducing a growth mindset resulted in enhanced attention to task-relevant stimuli (indicated by enhanced P3 and reduced late Pe), whereas inducing a fixed mindset enhanced attention to responses (indicated by reduced P3 amplitudes and enhanced late Pe amplitudes [15]
		- A higher growth mindset in math is marginally associated with a larger P300 response in response to negative feedback [21]
		- Students with fixed mindsets showed greater attention to positive feedback, as indicated by larger P300 amplitudes, especially in 4th graders. A marginal association between a fixed mindset and greater attention, as indicated by larger P300 amplitudes to negative feedback in 4th graders [22]
ERN	- Early detection of errors. - Conflict monitoring and automatic error detection. - Linked to error correction and behavioral adjustment. Time: 50–100 ms post-error.	- Mindset had no effect on ERN amplitudes [5,15,16,20]
Pe	- Conscious awareness of an error. - Error evaluation and cognitive control - Motivational aspect to correct behavior. Time: 400–700 ms post-error.	- Growth mindset was related to greater attention to mistakes in adults (larger Pe), and Pe mediated the link between growth mindset and post-error accuracy [5]
		- Growth mindset was related to greater attention to mistakes in kids aged 5–8 years old (larger Pe), but Pe did not mediate the link between growth mindset and post-error accuracy [16]
		- Inducing a growth mindset resulted in enhanced attention to task-relevant stimuli (indicated by enhanced P3 and reduced late Pe), whereas inducing a fixed mindset enhanced attention to responses (indicated by reduced P3 amplitudes and enhanced late Pe amplitudes [15]
		- Larger Pe amplitudes were initially observed in fixed-mindset individuals but disappeared after correcting for stimulus-response overlap [20]
FRN	- Response to negative feedback, reflecting disappointment or loss. - Linked to reward processing and the motivation to adjust behavior. Time: 200–400 ms post-feedback.	- No significant associations between FRN and mindset [21]
Left Temporal Negativity	- Memory-related activity during error correction or processing of feedback. - Reflects effortful encoding of corrective information. Time: 300–500 ms post-feedback	- Entity theorists showed reduced left temporal negativity after receiving corrective feedback, indicating less effortful encoding of corrective information [10]
N400, N600	- social expectancy violations Time: 400 and 600 ms post stimulus	- Entity theorists displayed a marginally greater N400 response to stereotype-violating information. In contrast, incremental theorists exhibited more pronounced N400 responses than entity theorists to stereotype-confirming information. No result was found for N600 [28]
α/β ratios	- Relax and stress index	- Intervention increased alpha wave activity and decreased high-beta wave activity during stress, indicating reduced stress response [25]
Prefrontal EEG asymmetry	- Related to motivation	- Significant larger frontal EEG asymmetry, indicating larger motivation for math mindset problems compared to standard problems [24]

**Table 5:** Information on neural measurements and related findings of included fMRI studies.

Neural Measurements	Main Neural Findings Regarding Mindsets
BOLD signals	- Fixed-mindset individuals exhibited stronger “punishment” responses to negative feedback in the caudate nucleus [19] - Growth mindset improvements were associated with increased activation and functional connectivity in the dorsal anterior cingulate cortex, striatum, and hippocampus [26]
Resting-state connectivity	- Growth mindset was associated with ventral and dorsal striatal connectivity with dACC and the DLPFC, which are considered important for error monitoring and concordant behavioral adaptation [12]
GMV	- A positive relationship between growth mindset scores and regional GMV of mOFC [11] - Association between higher grit and smaller rGMV in the left DLPFC and greater rGMV in the right putamen. Growth mindset acted as a mediator in the association between left DLPFC volume and grit [27]
